# Virulence Characteristics and Emerging Therapies for Biofilm-Forming *Acinetobacter baumannii*: A Review

**DOI:** 10.3390/biology11091343

**Published:** 2022-09-12

**Authors:** Karma G. Dolma, Rachana Khati, Alok K. Paul, Mohammed Rahmatullah, Maria de Lourdes Pereira, Polrat Wilairatana, Bidita Khandelwal, Chamma Gupta, Deepan Gautam, Madhu Gupta, Ramesh K. Goyal, Christophe Wiart, Veeranoot Nissapatorn

**Affiliations:** 1 Department of Microbiology, Sikkim Manipal Institute of Medical Sciences, Sikkim Manipal University, Gangtok 737102, Sikkim, India; 2 School of Pharmacy and Pharmacology, University of Tasmania, Hobart, TAS 7001, Australia; 3 Department of Biotechnology & Genetic Engineering, University of Development Alternative, Lalmatia, Dhaka 1207, Bangladesh; 4 CICECO-Aveiro Institute of Materials, University of Aveiro, 3810-193 Aveiro, Portugal; 5 Department of Medical Sciences, University of Aveiro, 3810-193 Aveiro, Portugal; 6 Department of Clinical Tropical Medicine, Faculty of Tropical Medicine, Mahidol University, Bangkok 10400, Thailand; 7 Department of Medicine, Sikkim Manipal Institute of Medical Sciences, Sikkim Manipal University, Gangtok 737102, Sikkim, India; 8 Department of Biotechnology, Sikkim Manipal Institute of Medical Sciences, Sikkim Manipal University, Gangtok 737102, Sikkim, India; 9 Department of Pharmaceutics, Delhi Pharmaceutical Sciences and Research University, New Delhi 110017, India; 10 Institute for Tropical Biology and Conservation, Universiti Malaysia Sabah, Kota Kinabalu 88400, Malaysia; 11 School of Allied Health Sciences and World Union for Herbal Drug Discovery (WUHeDD), Walailak University, Nakhon Si Thammarat 80160, Thailand

**Keywords:** *Acinetobacter baumannii*, biofilm, multidrug-resistance, microorganisms, antimicrobial, therapeutics

## Abstract

**Simple Summary:**

*Acinetobacter baumannii* (*A. baumannii*) is one of the ESKAPE organisms and has the competency to build biofilms. These biofilms account for the most nosocomial infections all over the world. This review reflects on the various physicochemical and environmental factors such as adhesion, pili expression, growth surfaces, drug-resistant genes, and virulence factors that profoundly affect its resistant forte. Emerging drug-resistant issues and limitations to newer drugs are other factors affecting the hospital environment. Here, we discuss newer and alternative methods that can significantly enhance the susceptibility to *Acinetobacter* spp. Many new antibiotics are under trials, such as GSK-3342830, The Cefiderocol (S-649266), Fimsbactin, and similar. On the other hand, we can also see the impact of traditional medicine and the secondary metabolites of these natural products’ application in searching for new treatments. The field of nanoparticles has demonstrated effective antimicrobial actions and has exhibited encouraging results in the field of nanomedicine. The use of various phages such as vWUPSU and phage ISTD as an alternative treatment for its specificity and effectiveness is being investigated. Cathelicidins obtained synthetically or from natural sources can effectively produce antimicrobial activity in the micromolar range. Radioimmunotherapy and photodynamic therapy have boundless prospects if explored as a therapeutic antimicrobial strategy.

**Abstract:**

*Acinetobacter* species is one of the most prevailing nosocomial pathogens with a potent ability to develop antimicrobial resistance. It commonly causes infections where there is a prolonged utilization of medical devices such as CSF shunts, catheters, endotracheal tubes, and similar. There are several strains of *Acinetobacter* (A) species (spp), among which the majority are pathogenic to humans, but *A. baumannii* are entirely resistant to several clinically available antibiotics. The crucial mechanism that renders them a multidrug-resistant strain is their potent ability to synthesize biofilms. Biofilms provide ample opportunity for the microorganisms to withstand the harsh environment and further cause chronic infections. Several studies have enumerated multiple physiological and virulence factors responsible for the production and maintenance of biofilms. To further enhance our understanding of this pathogen, in this review, we discuss its taxonomy, pathogenesis, current treatment options, global resistance rates, mechanisms of its resistance against various groups of antimicrobials, and future therapeutics.

## 1. Introduction

The superior capability of *A. baumannii* strains to produce biofilms correspondingly facilitates its colonization on surfaces, including medically useful instruments, indwelling catheters, and endotracheal tubes [1]. Biofilms are defined as an accumulated mixture of the microbial cells enclosed by an autogenic polymeric exopolysaccharide matrix which are produced on complex biotic or abiotic surfaces. Structurally, it forms a conglomerate system that defends microbial communities and renders an enhanced protective mechanism against several antimicrobial agents and host immune defense as well as harsh physiological parameters. Biofilm-forming bacteria are responsible for causing 65–80% of infections in humans (mainly chronic infections) [2]. Compared to other species, the rate of biofilm formation in *A. baumannii* is nearly 80–91%, whereas it is approximately 5–24% in the other species [3]. Furthermore, the biofilm formation has led to an increased resistance mechanism of these strains of bacteria against antimicrobial stressors and antibiotics. Thus, biofilm is represented as one of the potent virulence factors [4].

## 2. Factors Involved in Biofilm Formation

**Figure 1:**
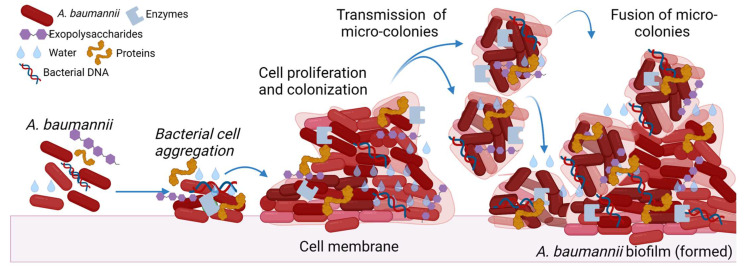
*A. baumannii* biofilm formation. The figure was made with www.biorender.com (accessed on 25 July 2022).

The process of biofilm production is dynamic [3]. Several studies have enumerated multiple factors responsible for the production and maintenance of biofilms. This includes physicochemical and microbial determinants such as the aggregation of substances, adhesion of collagen, expression of pili, capsular polysaccharides, and resistance determinants. Secretion of macromolecules, cell communication, and surface-regulated attachment are other essential factors in producing the biofilm. However, there are mainly three mechanisms of interaction that prime the establishment of biofilms—first, the interaction between the microbial cells. Second, bacterial adherence to the surface of human tissues or objects, and, finally, through serine lactones acylation, exchange of information occurs in the surrounding medium (Figure 1) [5]. The study performed by Maria-Guadalupe Avila-Novoa et al., reported that MDR *A. baumannii* strains exhibiting 100% resistance to several antibiotics in antimicrobial susceptibility tests have the potential to form biofilms in the clinical environment [6].

Excessive production of the matrix formed by exo-polymeric substances, biological heterogeneity due to physicochemical changes and persisters, and variations in bacterial phenotypic and genotypic countenance due to aggressive reactions of microbial aggregation are the factors causing impaired drug diffusion and, eventually, amplifying drug resistance in the phenotype of biofilm. Consecutively, extracellular polysaccharides (EPS) form one and several microcolonies, which fuse with each other and with the liquid channels connected to create a mature biofilm eventually. The biofilm formation by *A. baumannii* isolates is usually associated with the upregulation of genes such as metal ions, plasmids, transposons, integrons, and outer membrane protein expression [7]. Adherence is the preliminary criteria for forming the biofilm, and Csu pili facilitate the formation of biofilms on any abiotic surface in *A. baumannii* [8].

### 2.1. Physiological Factors

Several environmental conditions such as temperature, availability of oxygen, pH, and surface hydrophobicity directly regulate the formation of biofilms. Any changes in such conditions facilitate the communication of cells with each other via a process of the cell-mediated density gradient, which in turn triggers the formation of biofilms with typical phenotypic and genotypic features.

Various studies have observed diverse environmental niches in promoting biofilm formation in *A. baumannii* strains. For example, a study conducted by Marti et al. reported that an *Acinetobacter calcoaceticus*—*Acinetobacter baumannii* complex (ACB complex) forms the biofilms at the (solid-liquid and air-liquid interphases) [9]. In their study, the other ACB complexes, *A. baumaanii* strains, produced the highest biofilm at the air-liquid interface. Similarly, Tomaras et al. showed that the pilus formation and usher chaperone mediate biofilm formation in *A. baumannii* cells on abiotic surfaces. Another study by Yassine et al. found that pellicle formation at the liquid-air interface leads to biofilm formation [10].

The temperature has also been widely reported to significantly alter the ability of the bacterial cells to mediate their attachment and generate biofilms on the surface. The study demonstrated by Marti et al. showed the elevated amount of biofilm formation by the *Acinetobacter* spp. at temperatures from 25 °C to 37 °C [9]. In another study, Pour N et al. showed that an optimum temperature of 30 °C with a constant pH of 7.0 supplemented with sodium chloride medium (at a concentration of 5 g/L^−1^) was the most favorable temperature for the formation of biofilm by *A. baumannii* isolates [10]. An increase in biofilm production was observed by Wei X et al. at an air-liquid interface which was mediated by isolates belonging to the ACB complex [11].

### 2.2. Type and Nutrient Availability

Nutrient availability and concentration have a profound effect on the accumulation of biofilms. A high concentration of nutrients negatively impacts biofilm formation due to the dissolution and minimum competition amongst the bacterial strains, which is otherwise necessary for the aggregation and forming biofilms. The frequency of biofilm formation is enhanced by the availability of low levels of nutrients. The growing proportion of biofilm is always doubled progressively in the availability of supplements, including sucrose, calcium, and phosphate. Excessive control of the nutrient supplement often results in the decreased production of exopolysaccharides and depletion in the level of biofilm production [11].

### 2.3. Growth Surface

The growth surface is one of the predisposing factors contributing to biofilm formation. The surface granularity and irregularities provide a shield to the bacteria by protecting it against the shear forces that allot turnaround time for the permanent attachment and hence promote biofilm production. The organic surfaces composed of different chemicals and nutrients facilitate the greater bacterial adherence. In electroactive microbes, biofilm production is influenced by factors including electrical changes on the surface and surface hydrophobicity [12].

### 2.4. Iron Concentration

The concentration of iron and sources of iron produces strain on the biofilm formation. Bap (biofilm-associated protein) is usually upregulated by limited iron availability. Increased iron concentration enables an increased hindrance to some selective antibiotics via signaling or by interacting with the antibiotics themselves. Most of the bacteria release potent iron-chelating substances known as sidephores to scavenge the surrounding iron available. Once iron is encountered by sidephores, it eventually forms an iron–sidephores complex. It binds to the specific outer membrane receptors, and this facilitates easy passage of molecules across the outer membrane. However, when the iron is available in higher concentrations, sidephores are insufficient to form complexes and cannot form passages in the outer membrane, thereby inhibiting the antibiotics from diffusing to the outer membrane. [13].

### 2.5. Expression of the Gene Involved in Biofilms

Some studies have demonstrated that the formation of biofilms is induced by biological signals and regulated by gene expression in a closed system. The genes reported to participate in the adherence and the generation of biofilm in *A. baumannii* strains are generally *CsuC, CsuD, CsuE, OmpA, blaPER-1, abaI, epsA, bfin S&R*, six genes of pilus synthetic system and gene sequence ST 25 and ST 78 identified by Multi-Locus Sequence typing [12]. The genes involved in biofilm development are summarized in Table 1. The expression of int I 1 mRNA is also increased markedly during the formation of biofilm by *A. baumannii*. Two genes of class I integron and 16S RNA methylase genes together are involved in gene movement and spread, which enhances the high expression of biofilm-related gene sequence [13]. One of the ribonuclease protein families T2 enables biofilm formation in *Acinetobacter* spp. as it promotes the adhesion and motility of *A. baumannii*.

### 2.6. Virulence Factors Associated with Biofilms

There are multiple predisposing virulence factors that contribute to the formation of biofilms in the *A. baumannii* strain. It includes the outer membrane protein A (OmpA), biofilm-associated protein (Bap), chaperon-usher pilus assembly system of pili (Csu BABCDE), extracellular exopolysaccharide (EPS), two-component system (Bfm/S BfmR), poly-β-(1,6)-N-acetyl glucosamine (PNAG), PER-1 belonging to β-lactamase family, and the Quorum sensing system [21,22].

### 2.7. Outer Membrane Proteins

*Acinetobacter* species possess an outer membrane protein (OMP) that contributes to the pathogenicity and development of antibiotic resistance in an organism. The major factor that plays a key role in the bacterial pathogenesis of the bacteria is the presence of outer membrane proteins (OMPs) [23]. Porins act as an essential factor in microbial virulence. The bacteria can easily prevent the antimicrobial drugs from penetrating across the outer membrane channels with the help of porin. The most prominent porin in *A. baumannii* is OmpA, which acts by developing resistance to drugs, attaching to epithelial cells, and, thus, forming biofilms [24]. OmpA of *A. baumannii* (AbOmpA) is the most prominent surface protein with a molecular weight of 38 kDa that facilitates the transfer of small solutes [25]. It plays a significant role in displaying its function through attachment and attack in the epithelial cells with the help of fibronectin. These proteins also contribute to serum resistance, biofilm formation, persistence, induction of apoptosis, and development of antimicrobial resistance in *A. baumannii* [24]. OMPs enable cell membrane integrity and facilitate increased cell adhesion to the surfaces [26]. OMPs are essential for drug resistance and the production of biofilms as they modulate the formation of outer membrane vesicles [27]. OmpA causes apoptosis of eukaryotic cells. It progressively stimulates the dendrite cells, which eventually facilitates the differentiation of CD4T cells towards a Th1 phenotype, causing an immune escape [28].

### 2.8. Biofilm-Associated Protein

Bap (biofilm-associated protein) are proteins that are associated with the formation of biofilms. Bap is usually a higher molecular cell surface protein with a molecular weight of about (854-kDa) and 8620 amino acids residing on the surface of bacteria. The protein encoded by the Bap gene plays a significant role in the adhesion of intercellular cells, aggregation of bacterial cells, maturation, maintenance, and development of biofilm [29]. It also contains a core domain of tandem repeats, which flexibly provides bacteria with the tendency to produce a biofilm [30]. Loehfelm et al. demonstrated the presence of adhesion molecules of the bacterial Bap on the cellular surface of the Acinetobacter species for the first time in 2008. It was demonstrated as a highly conserved area, actively facilitating cellular adhesion and eventually leading to the maturation of biofilm in different substrata [31,32].

*Acinetobacter* spp. contains several different types of Bap proteins, all of which possess large central repetition regions. It is primarily composed of either 80–100 amino acids (aa) long repeat of Ig-like (alpha-type Bap or homopolymeric stretches of alternating amino acid (aa) residue (mainly the residues of serine and aspartic acid or serine and alanine) comprising nearly 500 dipeptides (β-types Bap). The alpha and beta-type Bap with repetitive region moves across the cell wall, mediating the exposure of the N-terminal region to the environment [33,34]. A study conducted by Brossard et al. revealed that the Bap is necessary for 3D tower structure and for aqueous channel formation on clinically important objects and areas, such as polypropylene, polystyrene, and titanium. The study also revealed the facilitation of enhanced hydrophobicity and adhesive property of the bacterial cellular surfaces by Bap protein [35]. Bap protein in *A. baumannii* is necessary for maintaining the stability of mature biofilms on glass surfaces, influencing both the biovolume and its thickness [36].

### 2.9. Chaperon-Usher Pilus Assembly System of Pili (Csu BABCDE)

Pili facilitate the adhesion and also generate the capacity to produce biofilm. The biofilm production on the abiotic surface by *A. baumannii* is mediated by Csu pili [37]. In *A. baumannii*, pilus-like bounded structures are formed from a clustered gene known as Csu operon. The Csu pilus is mainly composed of nearly four protein submits, Csu A/B, CsuA, CsuB, and CsuE, which function primarily through pathways known as archaic chaperone–usher (CU) pathways. The production of biofilms is based on the arrangement of the CsuA/B, CsuA, CsuB, CsuE, and CsuC-CsuD chaperone-usher secretion machinery, and the formation of pili is necessary for attachment to abiotic surfaces [38]. Among the entire CU system, archaic CU pili form a massive cluster of CU systems, and in combination with other substitutional CU families, it further forms a nonclassical branch of the CU superfamily [39]. Some relevant studies have demonstrated that the inactivation and deregulation of the *CsuE* gene abolish the formation of both pilus and biofilm. One of the prominent genes of the two-component system, namely, *bfmSR*, controls the expression of Csu operon. The *bfmSR* system mainly comprises the *bfmS* and *bfmR* gene [40]. *BfmS* gene is known as histidine sensor kinase that prominently identifies the environmental condition. *BfmRs* are the encoding genes that regulate the responses. A similar study conducted by Cerqueira GM related to a two-component system stated that the system termed GacSA displayed the regulated and controlled expression of Csu and indirectly impacted biofilm formation [41].

### 2.10. Extracellular Polysaccharides (EPS)

EPS play a leading role in the expansion and pathogenesis of biofilm. The main composition of EPS is alginate and antibiotics hydrolytic enzymes immobilized on biofilm, which functions by preventing the entry of antibacterial agents into the target and reducing the antibacterial activity [42]. EPS are mainly polysaccharides associated with a polypeptide chain that are negatively charged amino acid side chains that tend to attract the positively charged amino side chains. This force of attraction hinders the penetration of hydrophilic antibiotics into the cell bodies and considerably decreases the bactericidal ability. It is one of the most probable reasons why bacteria are not easily removed after biofilm formation. The multiresistant strain of *A. baumannii* has an O-glycosylation system and capsule synthesis with the major involvement of EPS [43].

### 2.11. Quorum Sensing (QS)

Quorum sensing (QS) is a series of events where microbes, mainly bacteria, exchange, sense, transport, and mediate active participation by releasing one or many chemical molecules of lesser molecular weight.

In *A. baumannii*, biofilm synthesis is controlled by the QS system caused by N-acyl-homoserine lactone (AHL) molecules which act as autoinducers. The information is mainly interchanged between bacteria through AHL molecules generated by a single bacterium which facilitates a huge accumulation of homologous bacteria. The massive number of intercellular adhesin (virulence factors) molecules in the polysaccharides are produced when the bacterial number reaches a critical level, and AHL becomes an effective sensing signal. When the population of bacteria reaches a certain level, biofilms are formed by embedding the microcolonies, and their production is facilitated by effective sensing of the signal by AHL [44]. However, Stacy et al. reported that with the help of the QS system, bacteria could possibly coordinate with each other as well as the different species to control their own behavior, and such interaction between different strains can lead to multiple infections [45]. The study by Liou et al. confirmed the involvement of another sensor in forming biofilms known as kinase BfmS. They demonstrated the significant reduction of biofilms in the absence of sensor kinase BfmS, which provided a new theory for further research on biofilm control [46]. The constrained iron supply enhances the expression of genes of the QS system that regulates the virulence factor based on the density of the bacterial species [47].

## 3. Current Antimicrobials for the Treatment of *Acinetobacter* Infections

**Figure 2:**
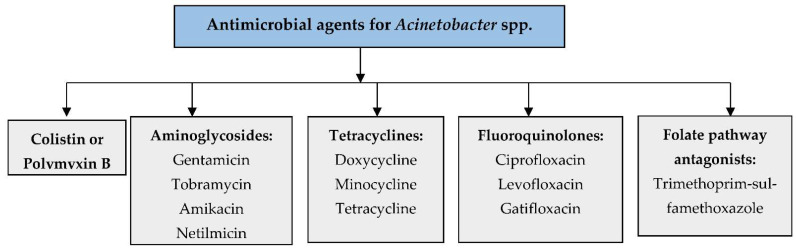
Antimicrobial agents used for *Acinetobacter* spp. infections.

The current treatment with antibiotics remains limited for *Acinetobacter* infections due to the increasing tendency to develop resistance to various antibiotics [48]. Generally, the non-*baumannii* species of *Acinetobacter* spp. are harmless to healthy individuals, but colonization led by *A. baumannii* can cause fatal infections in immunocompromised patients [49]. Therefore, the highly recommendable antibacterial agents for susceptible *A. baumannii* infections are mainly β-lactam antibiotics. Colistins, sulbactam coformulated with ampicillin, tigecycline, minocycline, carbapenems, piperacillin/tazobactam, cefepime, doxycycline, aminoglycosides, and quinolones are currently used as frontline antibiotics for the treatment of A. *baumannii* infections [50]. During Acinetobacter infections, the drugs generally used for the treatment are sulbactam, imipenem-cilastatin, meropenem, doripenem, amikacin tobramycin, colistin (colistimethate), polymyxin B, tigecycline, and minocycline. According to the CLSI guidelines 2022, the following lists of antimicrobial agents are used for *Acinetobacter* spp. infections (Figure 2) [51].

### Combination Therapy

The formation of biofilms increases the resistance of *Acinetobacter* spp. to antibiotics. Thus, one of the possible and effective ways to treat biofilm-associated infections could be combination therapy. Due to the suboptimal pharmacokinetics of the drugs and rapid emergence of resistance, combination drug therapy is attracting frequent attention for the treatment of such infection. Recently, combination therapy and monotherapy (e.g., amikacin, minocycline or colistin, rifampicin) are also proving to be effective against *A. baumannii* infection. An excellent review study was made by Petrosillo et al. on colistin versus combination therapy. They demonstrated the relevance of only four clinical studies with respect to mortality and showed that only one study favored monotherapy, demonstrating statistical significance [52]. A prospective noncomparative study conducted by Bassetti et al. in severely sick patients with pneumonia and bacteremia demonstrated a colistin–rifampicin combination [53]. An in vitro antagonistic study was performed on biofilm-embedded *A. baumannii* strains to measure the strength and potency of antibiotics such as colistin, tigecycline, and levofloxacin solely or in amalgamation with clarithromycin and/or heparin as lock solutions. Biofilm-embedded *A. baumannii* strain showed bactericidal activity when treated with a combination of clarithromycin [54]. Another in vitro study to compare the efficacy of combined drugs, including colistin–levofloxacin, colistin–tigecycline, and tigecycline–levofloxacin-based catheter lock solutions, was performed [55]. The most potent antibacterial activity was displayed by colistin–levofloxacin, though the other drugs in combination also demonstrated bactericidal activity but at a lower level.

Song et al. evaluated the strength of imipenem and rifampicin solely and in combination against clinical isolates of *A. baumannii* in biofilm and planktonic culture. However, they observed that there was no significant reduction in the biofilm formation at the MIC of each of the antimicrobial agents of imipenem, colistin, and rifampicin when used individually. It was observed that imipenem, colistin, or rifampicin did not show any susceptibility against *A. baumannii* biofilms [56]. Compared to the positive control, tigecycline imipenem–rifampicin and colistin–rifampicin displayed a considerable reduction in biofilm synthesis after 48 h of incubation. They evaluated that combination therapy could be potent for controlling and reducing biofilm formed by *A. baumannii* strains by using antibiotics including tigecycline, imipenem–rifampicin, and colistin–rifampicin. For carbapenem-resistant and carbapenem-susceptible *A. baumannii* biofilms, the combination therapy of sulbactam–tigecycline was reported to be effective as an alternative treatment [56,57].

## 4. Future Therapies for the Treatment of *Acinetobacter* Infections

**Figure 3:**
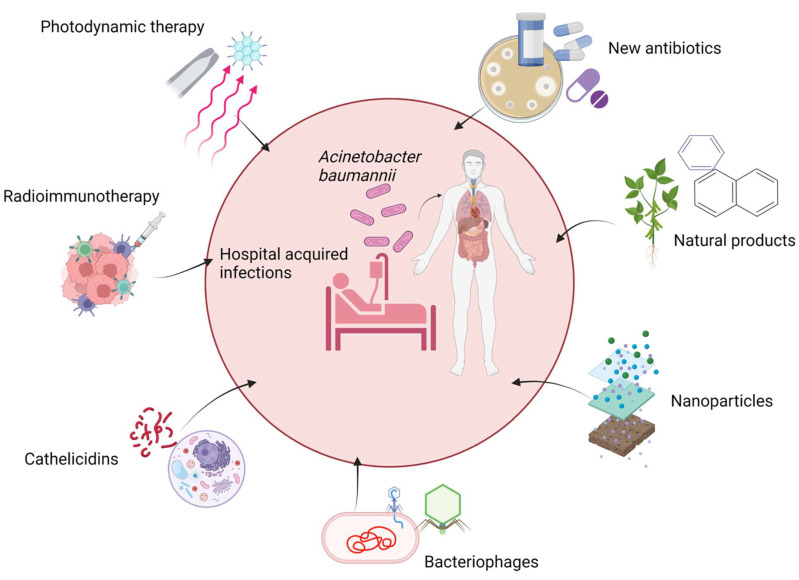
Possible future therapies for *A. baumannii* infection. The figure was made with www.biorender.com (accessed on 25 July 2022).

Numerous attempts have been made to develop alternative approaches with improved susceptibility to *Acinetobacter* spp. The future strategies which possibly could help to overcome the acquired resistance mechanism of antimicrobial agents are new antibiotics, natural products, nanoparticle technology, bacteriophage therapy, bactericidal gene transfer therapy, cathelicidins, radioimmunotherapy, and photodynamic therapy, as shown in Figure 3.

### 4.1. New Antibiotics

Existing management choices for carbapenem-resistant *A. baumannii* (CRAB) are restricted and demonstrate many pharmacokinetic boundaries. Even with the last resort of drugs such as tigecycline or colistin, the drug-resistant *A. baumannii* progressively poses a severe hazard to public well-being worldwide. There is an urgent need for investments in new drugs.

Multiple companies are performing clinical trials to treat *Acinetobacter* spp effectively. Many of these are at the preclinical stage or at a very early stage of development. Under the siderophore cephalosporins, the GSK-3342830 is an injectable cephem antibacterial that targets Gram-negative bacteria developed by GlaxoSmithKline where considerable activity against CRAB was demonstrated [58]. The GT-1 action, contrary to CRAB in vitro and in animal study, has shown promising results as carried out by Geom Therapeutics [59]. Cefiderocol (S-649266), another novel cephalosporin conjugated with a catechol siderophore on its side chain, has revealed effective antibacterial activity against the carbapenem-resistant strains of *A. baumannii* with MIC_50/90,_ 1–8 µg/mL [60]; however, it presented with less effective activity against the OXA-23 and OXA-40 [61]. Fimsbactin is a natural siderophore of *A. baumannii*; Ghosh M et al. [62] demonstrated in an in vivo study of mice that fimsbactin conjugate demonstrated good in vivo activity. Entasis Therapeutics has developed a drug that is a novel beta-lactamase inhibitor combined with sulbactam. It is a drug with a combination of Sulbactam and Durlobactam, which exhibited effective antimicrobial activity against clinical isolates of MDR strains of ABC complex [63].

Sulbactam-ETX2514, is a broad-spectrum diazabicyclooctanone (DBO) β-lactamase inhibitor. Hackel M et al. [64]’s study showed that it was active against 91% OXA carriers (MIC50/90, 1/4 µg/mL) and several colistin-resistant strains with an MIC of 2 µg/mL. Several trials are being conducted for investigating ETX2514 in combination with either sulbactam or imipenem-cilastatin, which have shown good results [65,66]. Another DBO β-lactamase inhibitor, the WCK 4234, was found to be effective against classes of carbapenemases as demonstrated in several studies [67,68]. The LN-1-255, was found to be active against class D-lactamases in vitro [69]. Under the new class of β-lactam antibiotics, FSI-1671 presented an efficient activity against CRAB [70]. Apramycin is an aminoglycoside. A study by Kang AD et al. [71] exhibited an MIC_50/90_ 8/32 µg/mL; MIC range, 2 to 256 µg/mL against several *A. baumannii* isolates.

Spero Therapeutics has developed SPR741, which is a polymyxin-derived potential peptide that is combined with other antibiotics used against the other Gram-negative bacteria, including *Acinetobacter.* It is effective in reducing the infections caused by *Enterobacteria*, including *Acinetobacter* [66]. Melinta is developing a novel ribosomal protein synthesis inhibitor that specifically has an effective role in inhibiting Gram-negative activity against *A. baumannii* [72]. Tetraphase Pharmaceuticals is developing eravacycline, a novel fluorotetracycline that has expanded activity against *Acinetobacter*. Eravacycline has completed phase III clinical trials for complicated intra-abdominal infections (cIAI) and complicated urinary tract infections (cUTI); more trials will likely be required based on unexpectedly low cure rates for cUTI. This drug may provide an excellent treatment option for *Acinetobacter* infections [73]. A study by Seifert and colleagues [74] showed that eravacycline (TP-434), a flurocycline (tetracyclines), MICs were found to have a greater activity as compared to tigecycline MICs against CRAB. In several other studies, the isolates that produced carbapenemase genes demonstrated MIC_50/90_, 0.5/1 µg/mL [75,76,77]. Another novel drug TP-6076 has also displayed good results against clinical CRAB [78] by inhibiting bacterial protein synthesis. LpxC, another aminoglycoside, is a zinc-dependent deacetylase. The MIC_50/90_ values of a new LpxC inhibitor were 0.8/3.2 µg/mL (MIC range, 0.5 to 64 µg/mL) when tried on clinical *A. baumannii* isolates [79]. The RX-P873 also demonstrated effective activity against *A. baumannii* isolates with MIC_50/90_ values of 0.5/1 µg/mL (MIC range, 0.12 to 4 µg/mL) [80].

### 4.2. Natural Products

With the rise of drug resistance observed in the late 1970s, there was a dearth of drugs against various diseases caused by microorganisms. By the late 1990s, the effective and operative drug left to us was carbapenem, which also joined the drug resistance assemblage and made treatment challenging. Subsequently, having no innovative growth of drugs to counter the carbapenem-resistant strains, it has become necessary to emphasize the use of traditional medicine. The secondary metabolites mainly account for the antimicrobial activity of plants. There are several studies using plant extracts for evaluating the antimicrobial effect against drug-resistant pathogens. Some of the plants and their active compounds are listed in Table 2.

Bulgecin A is a natural derivative of *P. mesoacidophila*. It acts as a lytic transglycosylase inhibitor and works synergistically with β-lactams. Bulgecin A could be used as an adjunctive compound to enhance the life of carbapenems against *A. baumannii* infections. It is accomplished by resisting the growth of carbapenem-resistant *A. baumannii* strains, thereby restoring the antimicrobial activity of meropenem. Likewise, farnesol is another natural product derived from *Candida albicans* applied for quorum-sensing, distorting the integrity of the cell membrane of *A. baumannii*, changing the cellular morphology, and, thereby, enhancing the susceptibility of MDR *A. baumannii* strains to colistin [75]. Several active compounds produced from herbs have strong antimicrobial activities against various bacteria, including carbapenem-resistant *A. baumannii* [90]. Similarly, oleanolic acid present in food, medicinal herbs, and several plants displays a potent antibacterial activity against many pathogenic bacteria as it contains a triterpenoid compound. In a study conducted by Shin et al., oleanolic acid enhanced aminoglycoside uptake by altering membrane permeability and energy metabolism in *A. baumannii* [91].

### 4.3. Nanoparticle Formulation

In nanotechnology, the nanoparticles of metals such as silver, gold, platinum, etc. have effective antimicrobial actions against microorganisms. These metals have demonstrated encouraging results in the field of nanomedicine and have exhibited both antibacterial and antifungal activities. This technology is novel, inexpensive, and easily approachable as it produces a topical class of antimicrobials. It has proved to be efficient for treating intricate cutaneous infections, including those caused by *A. baumannii* strains [92]. In another study, it was observed that attachment of silver nanoparticles to *Acinetobacter* was not only effective against several multidrug-resistant organisms but expressively lessened the biofilm activities of these drug-resistant organisms [93]. Nitric oxide (NO) has been demonstrated to display an effective antimicrobial activity as well as a potent immune modulator regulating wound healing. Friedman et al. [94] made a stable nitric oxide (NO)-releasing nanoparticle (NO-NPs) by using nanotechnology-based silane hydrogel. Mihu et al. [92] have demonstrated the efficacy of NO-NPs against *A. baumannii* using a murine wound and soft tissue model. In the study, NO-NP-treated mice showed considerable deductions in bacterial encumbrance in comparison to control animals, thereby increasing the rate of wound healing and reducing collagen degradation by bacterial collagenases. Helal et al. showed the action of silver nanoparticles (AgNPs) against *A. baumannii* isolates. They treated the bacteria with AgNPs, which substantially disrupted the bacterial growth and proliferation. The virulent and biofilm-related genes were downregulated by the AgNPs at the transcriptional level, which led to the interruption in the bacterial growth [95]. A previous study reported that the usage of silver nanoparticles inhibited the MDR *A. baumannii* and further prevented the colonization and formation of biofilm on the human lung epithelia with less toxicity [96]. Hemeg et al. published the concordant antibacterial effects of Ag, Au, and ZnO NPs against Gram-positive and Gram-negative bacteria. They deduced that NPs act by entering the bacterial cell membrane and interrupting the crucial molecular pathways, evading antimicrobial mechanisms. They also showed that NPs combined with several antibiotics, including polymyxin B, ciprofloxacin, ceftazidime, ampicillin, clindamycin, vancomycin, or erythromycin enhanced the antimicrobial effect and successfully worked against the MDR strains of bacteria including *Acinetobacter* [97]. Based on the study by Chen et al. [98], they determined that AgNPs can simultaneously induce apoptosis and inhibit new DNA synthesis in multidrug-resistant *A. baumannii* in a concentration-dependent manner using three different methods such as colony-forming unit (CFU) method, flow cytometry (FC), and a BrdU ELISA. (Biomedical). Another study by Banoub et al. [99] demonstrated the significant in vitro activities of chitosan nanoparticles either alone or in combination with various antibiotics against the MDR *A. baumannii* pathogens. Moreover, the use of chitosan is recommended as they are biodegradable polymers and are nontoxic. Wan et al. proved that upon taking AgNPs with antibiotics to treat carbapenem-resistant *A. baumannii,* there was complete inhibition of *A. baumannii* [100]. The AgNP treatment also presented synergistic effects with the antibiotics polymixin B, rifampicin, and tigecyline. Hence, based on the efficacy of the nanoparticles in various studies, we can include nanotechnology as an effective approach to combat the infections caused by biofilm-forming bacteria.

### 4.4. Bacteriophage and Bactericidal Gene Transfer Therapy

Currently, the research area revolving around antibacterial phage therapy has gained a considerable interest. To counteract the phenomenon of antibiotic resistance, bacteriophage therapy is being re-evaluated as a substitutional treatment because of its high specificity and efficient role. Bacteriophages are viruses that invade and kill target bacteria by lysis. These phages are specific for different bacteria. They are known to bind to the specific receptors present on the cell walls of the bacteria [101]. They introduce the deoxyribonucleic acid into the cell and, in that process, lyse the cell. Various studies have been conducted with lytic bacteriophage therapy to combat drug resistance issues [102,103]. Schooley et al. demonstrated that phages were used on MDR *A. baumannii* pancreatic pseudocyst infection, resulting in a cure of the infection and complete clinical recovery [104]. In fact, recently, a study conducted by Yang et al. showed the efficacy of virulent AB1 bacteriophage against *A. baumannii* by isolating and characterizing virulent AB1 bacteriophage representing it as a unique therapeutic with some potent efficacy [105]. The amalgamation of phage-vWUPSU, family Myoviridae and sacha inchi oil as antimicrobial agents suggestively repressed and detached biofilms, compared with the effects of either single treatment [106]. In recent years, the antibacterial and anti-biofilm activities of several phages targeting MDR *A. baumannii* have been characterized [107]. Some of the phages are listed as: FAB1- and phage Abp2-specific MDR *A. baumannii* [108], Phage ISTD [109], Phage IsfAB78 [110], and Phage B-R2096 [111]. Another possible future therapy includes-bactericidal gene transfer therapy. Bactericidal gene transfer therapy is the process of representation and incorporation of vectors possessing the genes of bacteria into pathogenic recipient organisms by the process of conjugation using attenuated donor cells. Despite the limitation in the therapeutic potential, the necessity of incorporation of donor cells to the pathogen (to facilitate vector transfer) resulted in murine burn infection models, which showed positive effects of bactericidal gene transfer. A similar approach was taken by Ebrahimi et al., who demonstrated the efficacy of bactericidal genes in mice treated with a single dose of 1010 CFU of donor cells. They reported lower levels of *A. baumannii* in burn wounds than in untreated mice [112].

### 4.5. Other Products

Gallium is used as one of the treatment strategies to combat the biofilms formation in *Acinetobacter* species. Gallium is a group 13 semimetallic element. It is the element that participates in iron-binding sites of the chelators and proteins. Gallium binds to biological complexes containing Fe^3+^ and significantly destroys a vital redox-driven biological process [113]. Gallium is generally used either in complex form with inorganic compounds or as a simple inorganic or organic salt. Studies have reported that gallium nitrate or gallium protoporphyrin IX can probably be a potent therapeutic choice for treating MDR *A. baumannii* [114,115]. The D-amino acids have verified that D-His and D-Cys interrupt the biofilm formation, adherence, and advance proliferation of eukaryotic cells in *A. baumannii* [116].

Probiotics can be utilized to protect the host from MDR *A. baumannii* pathogens. Probiotics are “protective live microorganisms which when administered sufficiently can provide a potent health benefit to the host” [117]. Asahara et al. demonstrated the potential of probiotic (*Bifidobacterium breve*) to provide protection against MDR *A. baumannii* infections in the intestine [118]. The use of probiotics and immunomodulators, such as lysophosphatidylcholine [119], can decrease the severity of infection caused by *A. baumannii.* In addition, macrolide antibiotics such as clarithromycin can be used in combination with other antibiotics such as colistin, tigecycline, or imipenem, which could potentially reduce infection [119].

### 4.6. Cathelicidins

Antimicrobial peptides (AMPs) are an evolutionarily conserved, heterogeneous group of short oligopeptides produced by the innate immune system and shown to have broad-spectrum bactericidal activity against pathogens, including viruses, bacteria, and parasites, serving as an integral part of the immune system’s first line of defense [120]. Multiple AMPs have been isolated from natural sources, and many others have been synthetically produced [121,122]. They demonstrate antimicrobial activity in the micromolar range, and, compared with traditional antibiotics, kill bacteria very rapidly [120,122]. Cathelicidins are the antimicrobial polypeptides (AMPs) identified from prokaryotic to eukaryotic kingdoms, including bacteria, fungi, plantae, and animalia composed of an N-terminal signal peptide (about 30 amino acids), a highly conserved cathelicidin domain (99–114 residues) between signal peptide and mature peptide and a C-terminal mature peptide (12–100 residues) with diverse structures (sequence and length) and functions [121,122]. In humans, the only cathelicidin studied is human LL-37; it displays both antitumor and anti-HIV activity. The cathelicidin providing effective results against *Acinetobacter* species is Tammar Wallaby cathelicidinWAM1, and the action of WAM1 against bacterial pathogens is 3–80 times more potent than LL-37. WAM1 can be used parenterally in humans with enhanced potential as it is nonhemolytic against human red blood cells. Indeed, for future in vivo studies, WAM1 can be considered one of the potential candidates due to its ability to tolerate high salt concentrations and antimicrobial activity [123].

As they have strong bactericidal properties without significant toxicity, several cathelicidin-derived peptide antibiotics have been tested in clinical trials. Some of the cathelicidins used for the treatment are given in Table 3:

### 4.7. Radioimmunotherapy

Clinically, though this method has not been explored as a therapeutic antimicrobial strategy, such as in cancer cells, radioimmunotherapy has much potential and can target microorganisms rapidly and powerfully [131]. The principle of the technique is based on the specificity of antigen–antibody interactions. Radionuclide are delivered and release a lethal dose of cytotoxic radiation directly to the target cell leading to the lysis of the targeted cell. Radioimmunotherapy has effectively opted for the treatment of bacterial, fungal, and viral infections. As in in vitro studies, it has produced only temporary hematological toxicity in experimental animals. Based on the production of antibodies against *A. baumannii*, radioimmunotherapy can be opted as a novel therapeutic strategy that could possibly be used to treat infections caused by *A. baumannii* [132].

### 4.8. Photodynamic Therapy

Photodynamic therapy is a process that successfully combines nontoxic photosensitizers (PSs) with oxygen. These PSs, in combination with oxygen, visibly generate a reactive oxygen species (ROS), which oxidizes biomolecules, thereby lysing the infected cells. The application of photodynamic therapy (PDT) involves the treatment of localized bacterial infections by its topical application into the infected tissue, followed by illumination with red (or near-infrared) light with the potential of penetrating the infected tissue [133]. The experimental study conducted on the murine burn wound model demonstrated the efficacy of this technique against *A. baumannii*, displaying no harmful effects on wound healing. Recently, Tsai et al. conducted a study to investigate the increasing efficacy of PDT against many resistant pathogens such as *Acinetobacter* by making use of polycationic biopolymer chitosan. The study displayed a bactericidal effect on a 2–4 log scale, the complete eradication of bacteria within 30 min in the subsequent chitosan treatment (0.025%) combined with hematoporphyrin-PDT (initial inoculation of 108 CFU/mL). The considerable antimicrobial activity was not displayed by chitosan alone without prior PDT; rather, the effective response of chitosan was caused due to induction by PDT [134].

## 5. Conclusions

*A. baumannii*, being an opportunistic nosocomial pathogen, gained advert importance due to its emerging multidrug-resistant characteristics caused by several virulence factors, including biofilm formation. It has been estimated that the formation of biofilm renders bacteria more resistant to antibiotics compared to other free-living cells [135,136,137,138]. The factors that initiate the formation of biofilm have been well-understood by the main interaction between the ambient environmental factors and the bacterial cells [138,139,140]. In hospital surroundings, mainly in ICUs, *A. baumannii* currently exists as a potent drug-resistant bacterium. Despite several comprehensive studies, the pathogenesis and toxicity of *A. baumannii* strains still remain vague. The extensive studies on antibiotics and combination therapy direct the necessity for profuse studies that ascertain the pharmacodynamics of antibiotics in monotherapy and combination therapy [141]. The multi-drug resistance capability of *A. baumannii* needs comprehensive research to understand its overall resistance mechanism. Newly developed antibiotics can be useful to manage multidrug-resistant *A. baumannii* [142]. The study primarily focuses on the emergence of biofilms, their process of formation, biomarkers, and specific determinants of their EPS matrix. Study is also required so that effective and potent antibiofilm drugs can be produced. Thus, more insightful research should be performed soon to anticipate the pathogenesis of *A. baumannii* and perform a thorough study on the formation of biofilm and its virulence determinants so that it can lay the cornerstone for the development of potent antibiotics.

## Figures and Tables

**Table 1:** Gene involvement in biofilm formations.

Genes	Role and Functions	Authors
*CsuC* *CsuD* *CsuE*	Mediates pili-biogenesis and biofilm formation on abiotic surfaces	Wright et al., 2017 [14]
*OmpA*	Maintains the integrity of the cell membrane and the ability of cell adhesion to the cellular surface. Involved in the antibiotic efflux system.	Rumbo et al., 2013 [15]
*blaPER-1*	Encodes the PER-1 extended-spectrum b-lactamase and facilitates beta-lactamase resistance mechanism. Leads to antibiotic resistance.	Rodriguez et al., 2006 [16]
*abaI*	Encodes for an autoinducer synthase that produces the Quorum sensing molecule. Controls the factors, including biofilm synthesis and its surface movement.	Badave et al., 2015 [17]
*Bap*	Encodes the biofilm-associated protein (Bap). Maintains a three-dimensional structure tower and water channel for the movement of water in the process of synthesis of biofilms.	Cao et al., 2014 [18]
*epsA*	Codes for a capsular polysaccharide protein. Role is to export outer membrane protein.	Russo et al., 2009 [19]
*bfmS&R*	Gene encoding S-histidine sensor kinase and R-response regulator Initiates the synthesis of pili for biofilm attachment and formation on polystyrene surfaces.	Liou et al., 2014 [20]

**Table 2:** List of natural products used against *A. baumannii*.

Plant’s Name	Active Compounds	References
*Lythrum salicaria*	Hexahydroxy diphenoyl ester vescalagin	[81]
*Rosa rugosa*	Ellagic acid	[82]
*Terminalia chebula*	Terchebulin, Chebulagic acid, Chebulinic acid, Corilagin	[82]
*Scutellaria baicalensis*	Norwogonin, Baicalin, Baicalein	[82]
*Syzygium aromaticum*	Eugenol	[83]
*Cinnamomum zeylanicum*	Trans-cinnamaldehyde	[84]
*Oreganum vulgare*	Carvacrol	[83,84]
Green tea *Camellia sinensis*	Epigallocatechin gallate (EGCG)	[85]
	Epicatechin	[86]
	Theaflavin	[86]
*Lyciumchinense* Mill.	(+)-Lyoniresinol-3 alpha-O-beta-D-glucopyranoside	[87]
*Paeonia suffruticosa* Andr.	Paeonol	[87]
*Coptidischinensis* Franch.	Berberine	[87]
Green tea (*Camellia sinesis*)	polyphenol, (–)-epigallocatechin- 3-gallate (EGCG)	[88]
*Pantoea agglomerans*	*Pantoea* Natural Product 3 (PNP-3).	[89]

**Table 3:** Cathelicidins used for the treatment of *A. baumannii*.

Peptide	Structure	Activity	References
LL-37 (Human cathelicidin)	α-helical	Immunomodulation characteristics, broad-spectrum microbicidal activities. Demonstrated MIC of 16–32 μg/mL against *A. baumannii* inhibited and dispersed the *A. baumannii* biofilm in abiotic surfaces at 32 and 64 μg/mL.	[124,125]
SAAP-148	α-helical AMP	Eliminate acute and biofilm-related; inhibit the growth of *A. baumannii* MDR at a concentration of 6 μg/mL.	[126]
ZY4 cathelicidin-BF-15	Cyclic peptide	Good activity against *A. baumannii*, including standard clinical MDR strains with MIC values ranging between 4.6 and 9.4 μg/mL.	[127]
*Naja atra* cathelicidin (NA-CATH)	α-helical structure at N-terminal and an unstructured segment at C-terminal	Antimicrobial activity through the membrane lysis by membrane thinning or transient pore formation and inhibiting the bacterial growth at a concentration of 10 μg/mL.	[127,128]
AM-CATH36: two fragments AM-CATH28 and AM-CATH21	Found in American alligator	Inhibit the growth of both drug-resistant and sensitive *A. baumannii* at the 2.5 μg/mL concentration.	[129]
BMAP-27 BMAP-34 mCRAP	Mammalian cathelicidins	It quickly disrupts the bacterial cellular integrity. It has potent action of inhibition against biofilms and exhibit immunomodulatory function.	[130]
